# Yes‐associated protein promotes early hepatocyte cell cycle progression in regenerating liver after tissue loss

**DOI:** 10.1096/fba.1023

**Published:** 2018-11-27

**Authors:** Christoph Tschuor, Ekaterina Kachaylo, Udo Ungethüm, Zhuolun Song, Kuno Lehmann, Patricia Sánchez‐Velázquez, Michael Linecker, Patryk Kambakamba, Dimitri A. Raptis, Përparim Limani, Dilmurodjon Eshmuminov, Rolf Graf, Amedeo Columbano, Bostjan Humar, Pierre‐Alain Clavien

**Affiliations:** ^1^ Laboratory of the Swiss HPB and Transplantation Center, Department of Surgery University Hospital Zürich Zürich Switzerland; ^2^ Department of Biomedical Sciences University of Cagliari Sardinia Italy

**Keywords:** cell cycle entry, hepatomegaly, liver function, organ size control, reconstitution, replication, small‐for‐size syndrome, YAP

## Abstract

The ability of the liver to restore its original volume following tissue loss has been associated with the Hippo‐YAP1 pathway, a key controller of organ size. Yes‐associated protein 1 (YAP1)—a growth effector usually restrained by Hippo signaling—is believed to be of particular importance; however, its role in liver regeneration remains ill‐defined. To explore its function, we knocked down YAP1 prior to standard 70%‐hepatectomy (sHx) using a hepatocyte‐specific nanoformulation. Knockdown was effective during the major parenchymal growth phase (S‐phase/M‐phase peaks at 32 hours/48 hours post‐sHx). Liver weight gain was completely suppressed by the knockdown at 32 hours, but was reaccelerated toward 48 hours. Likewise, proliferative markers, *Ccna2/b2* and YAP1 target gene expression were downregulated at 32 hours, but re‐elevated at 48 hours post‐sHx. Nonetheless, knockdown slightly compromised survival after sHx. When assessing a model of resection‐induced liver failure (extended 86%‐hepatectomy, eHx) featuring deficient S‐ and M‐phase progression, YAP1 was not induced at 32 hours, but upregulated at 48 hours post‐eHx, confirming its dissociation from M‐phase regulation. Therefore, YAP1 is vital to push hepatocytes into cycle and through the S‐phase, but is not required for further cell cycle progression during liver regeneration. The examination of YAP1 in human livers suggested its function is conserved in the regenerating mammalian liver.

**Abbreviations**
ALKP: alkaline phosphatase
ALT: alanine transaminase
AST: aspartate transaminase
eHx: extended 86%‐hepatectomy (inducing liver failure/SFSS)
LDH: lactate dehydrogenase
LW/BW: liver‐weight‐to‐body‐weight ratio
pH3: phospho‐histone 3
SFSS: small‐for‐size syndrome
sHx: standard 70%‐hepatectomy
YAP1: Yes‐associated protein 1

## 1 INTRODUCTION

The vital functions of the liver require steady maintenance. Consequently, the liver is equipped with a unique regenerative capacity and can regrow its original mass following extensive tissue loss. After surgical resection, the human liver regains normal function within 2‐3 weeks, while liver volume is completely restored after a few months.^1^ In mouse, partial 70%‐hepatectomy (sHx, the standard model of liver regeneration) induces efficient regeneration leading to a complete volume regain within a week.^2^

The reconstitution of the exact liver volume following tissue loss points to an optimal *liver‐size*‐to‐*body‐size* ratio required for robust long‐term function. On the other hand, the liver continues to provide key functions also after tissue loss. If tissue loss, however, is too extensive, the liver remnant loses the ability to regrow, leading to liver failure and eventually death. In mouse, massive resection (ie, 90%) of the liver causes 100% mortality within 2 days, while mortality is at ~30% following extended 86%‐hepatectomy (eHx), with survivors displaying metabolic liver dysfunction (hyperbilirubinemia, hypoalbuminemia, persisting steatosis) and a proliferative arrest in hepatocytes.^2^, ^3^ In humans, resection‐induced liver failure is known as the small‐for‐size syndrome (SFSS), likewise features metabolic as well as regenerative insufficiency,^3^ and is the most frequent cause of postoperative death.^4^ Therefore, liver size is a key determinant of liver function, with an optimal volume for sound function and a minimal volume to sustain vital function under stress.

The most prominent pathway regulating organ size is the Hippo‐YAP1 axis. In brief, the MST1/2 and downstream LATS1/2 kinases are the conserved regulators of yes‐associated protein 1 (YAP1) to restrain its activity via phosphorylation‐induced degradation. Upon triggers such as the loss of cell‐cell contact, disturbed cell polarity, or mechanical stress, MST1/2‐LATS1/2 activities decline, and so does proteasomal degradation of YAP1. Accumulating YAP1 then can translocate to the nucleus where it acts—in concert with TAZ—as a transcriptional coactivator to promote the expression of its target genes such as *Birc5* or *Ctgf*.^5^ The release of YAP1 activity usually is associated with cell cycle entry and apoptotic suppression resulting in an enlarged organ.^5^

In the liver, the transgenic overexpression of YAP1 or liver‐specific deletion of MST/LATS causes hepatomegaly that, however, is reversible upon YAP1 inactivation.^5^, ^6^ Furthermore, cholestatic injury in YAP1‐deficient mouse liver is associated with excessive necrosis,^7^ consistent with a proregenerative function of the protein. In mouse liver following sHx, YAP1 but not TAZ activity (ie, nuclear expression and target gene expression) increases while MST/LATS activities decrease, followed by a return to baseline levels upon completion of regeneration.^7^, ^8^ Elevated MST/LATS activities have further been found to be responsible for the regenerative deficits of aged liver.^9^ Besides its generic control through the Hippo pathway, YAP1 is also promoted through hedgehog signaling during regeneration, with hedgehog inhibition impacting both hepatocyte proliferation and YAP1 activation.^10^ In a recent report, liver‐specific YAP1 knockout was associated with reduced Ki67 counts at 48 hours after hepatectomy^11^; however, no further proliferative parameters or time points were analyzed.

Collectively, the above findings suggest YAP1 activity is required after tissue loss for a liver to regenerate. However, available evidence remains largely associative or is insufficient to provide clear insight into the role of YAP1 in the regenerating liver. To appreciate the function of YAP1 in parenchymal regeneration, its acute loss in hepatocytes may be a suitable strategy as to minimize compensatory mechanisms or pre‐existing pathologies.^12^, ^13^ To this end, we used a nanoformulation to achieve hepatocyte‐specific knockdown of YAP1 in a mouse model of standard hepatectomy. Moreover, we compared outcomes with an established model of resection‐induced liver failure and validated findings in human liver tissue.

## 2 MATERIALS AND METHODS

### 2.1 Animals

Animals aged 8‐10 weeks were kept on a 12‐hour day/night cycle with free access to standard chow and water. Littermates were housed in ventilated cages with automated ambient regulation at room temperature in an environment enriched with paper and bedding for nesting. Male wild‐type mice (C57BL6, Envigo, Horst, NL) were used.

### 2.2 Animal surgery

Standard hepatectomies (sHx, 70%, fully regenerating, 100% survival) and extended hepatectomies (eHx, 86%, regenerative delay, ~70% survival, SFSS model) were performed as reported.^2^ Sham operation consisted of cholecystectomy. The gain in liver weight, a physical measure of liver regeneration, was expressed through the ratio of liver‐weight‐to‐body‐weight (LW/BW).

### 2.3 Yap1 knockdown

siRNAs targeting *Yap1* and the control *Ahsa1* (ie, without regenerative function) were designed by Axolabs Gmbh (Kulmbach, D) and packed into company‐owned formulations designed to preferentially target murine hepatocytes. Formulations were injected into the tail vein 48 hours before hepatectomy. The lack of significant toxicity was ascertained through the assessment of liver injury markers.

### 2.4 Immunochemistry

Immunostainings were performed on 3‐μm formalin‐fixed, paraffin‐embedded liver sections. Antigenes were retrieved by boiling in citrate buffer. The following primary antibodies were used: Ki67 (Abcam, Cambridge, UK; ab16667), pH3 (Abcam; ab92628), YAP1 (Santa Cruz, Santa Cruz, CA, USA; sc‐15407), PCNA (Abcam; ab29). Secondary detection was done using the Ventana Discovery automated staining system and the iView DAB kit (Ventana Medical Systems, Basel, CH). Blinded counts were from 10 random fields (20× magnification) per sample. Biopsy tissue was obtained from hepatectomy patients without postoperative complications (n = 7, normally regenerating liver, retrieved at day 7 (3), day 8 (1), day 9 (2), and day 11 (1) postsurgery), and from hepatectomy patients that had developed SFSS (n = 7, SFSS, retrieved at day 5 (1), day 7 (1), day 9 (2), day 10 (1), day 12 (1), and day 14 (1) postsurgery). The clinical diagnosis of SFSS was based on the “50‐50 criteria.”^14^

### 2.5 Quantitative real‐time polymerase chain reaction

Total RNA was extracted from 20 mg of liver tissue using TRIzol reagent (Invitrogen, Zug, CH) and transcribed into cDNA using the ThermoScript reverse‐transcription PCR System (Invitrogen). TaqMan gene expression assays for *Ccnd1* (Mm00432359_m1), *Ccne1* (Mm00432367_m1), *Ccna2* (Mm00438064_m1), *Ccnb2* (Mm01171453_m1), *Yap1* (Mm01143263_m1), *Ctgf* (Mm01192932_g1), *Birc5* (Mm00599749_m1), *Myc* (Mm00487803_m1), *Cyr61* (Mm00487498_m1), and 18S rRNA internal control (TaqMan ribosomal RNA control reagents) were from PE Applied Biosystems (Rotkreuz, CH). The results represent fold induction (2^−∆Ct^) of mRNA expression ±SD.

### 2.6 Serum measurements

Serum samples were obtained from the inferior vena cava before organ harvesting. Albumin, bilirubin, ALT, AST, ALKP, and LDH levels were measured using a serum multiple biochemical analyzer (Dri‐Chem 4000i, Fujifilm, Dielsdorf, CH).

### 2.7 Western blotting

Western blot was performed as described previously.^2^ The following primary antibodies were used: YAP1 (Cell Signalling, Beverly, MA, USA; 4912) and GAPDH (Abcam ab9484).

### 2.8 Statistical analysis

Data are presented as mean ± SD. Differences between the groups were assessed by a two‐tailed *t* test assuming unequal variance. At least 5 mice/group were analyzed unless otherwise stated. For the molecular analyzes following siRNA knockdown, ≥ 3 mice/group were included. Differences were considered significant at *P* < 0.05 and indicated in figures by an asterisk (*). Statistical analyzes were performed using Prism 6.0 (GraphPad, La Jolla, CA).

### 2.9 Study approval

All animal experiments were in accordance with Swiss Federal Animal Regulations and approved by the Veterinary Office of Zurich. Ethical approval for the human sections was granted by the regional ethics committee (KEK‐ZH‐Nr 2012‐01 08). A written consent to study tissue for research purposes was received from the patient prior to inclusion in the study.

## 3 RESULTS

### 3.1 Hepatocellular YAP1 is activated during the major proliferative wave after resection

**Figure 1:**
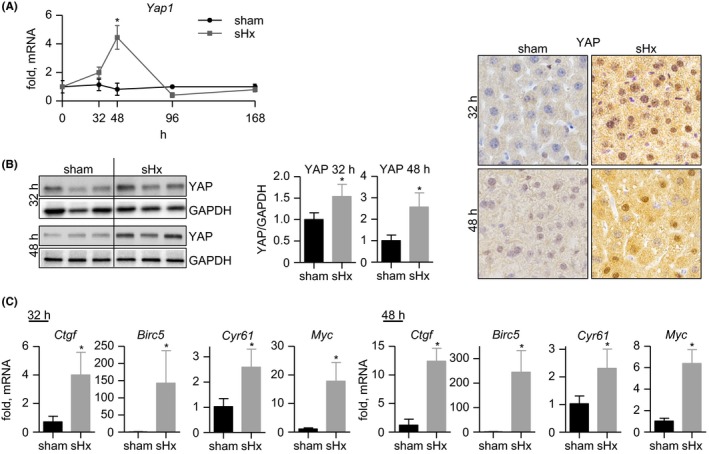
YAP1 is induced during the major parenchymal growth phase after hepatectomy. (A) *Yap1* mRNA expression after sHx or sham surgery. (B) YAP1 protein expression at 32 hours and 48 hours after sHx/sham assessed by immunoblots and immunohistochemistry. Note the nuclear accumulation after sHx. (C) YAP1 target gene expression after sHx/sham. N = 5/group, *t* test, *P < 0.05

YAP1 has been reported to be upregulated in liver after tissue loss.^8^, ^10^ To confirm its elevation in our model of standard 70%‐hepatectomy (sHx), we assessed hepatic *Yap1* mRNA at various times after resection. Relative to sham operation, *Yap1* expression rose at 32 hours (*P* = 0.053) and 48 hours (*P* < 0.05) post‐sHx, and declined again at 96 hours to baseline levels (Figure 1A). Immunoblots confirmed YAP1 protein upregulation at 32 hours and 48 hours (Figure 1B), the usual S‐ and M‐phase peaks after sHx.^8^ Increased protein levels were accompanied by nuclear translocation of YAP1 (Figure 1B), and an elevated expression of its transcriptional targets *Ctgf*,* Birc5*,* Cyr61*, and *Myc* relative to sham controls (Figure 1C). Therefore, YAP1 appears to be activated during the major growth phase of the regenerating mouse liver after resection.

### 3.2 YAP1 knockdown impairs liver regeneration at 32 hours but not 48 hours after hepatectomy

**Figure 2:**
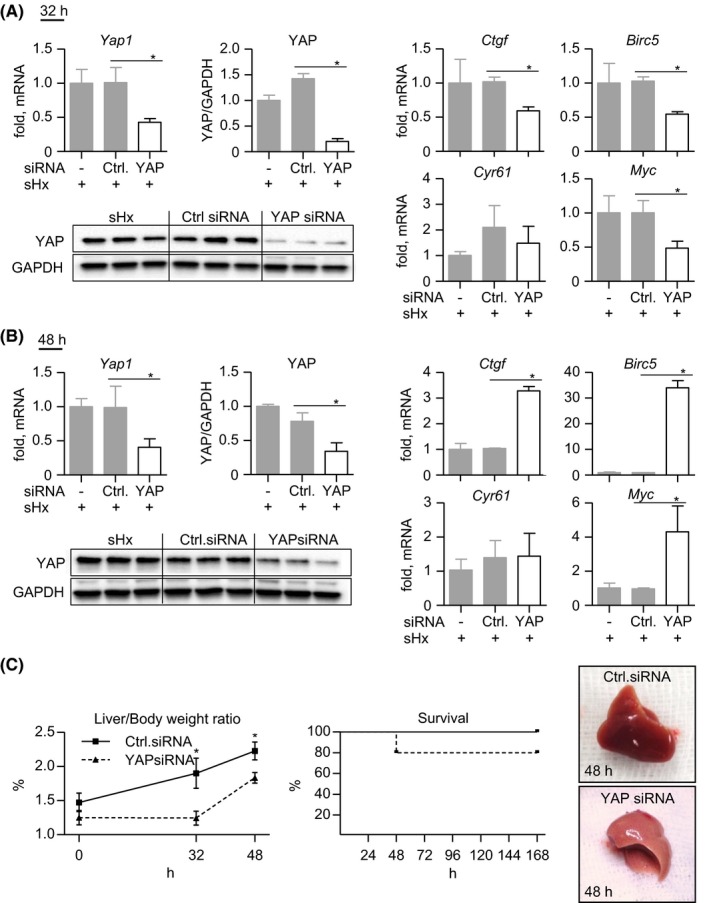
*Yap1* knockdown impairs liver regeneration up to 32 hours following sHx. (A) *Yap1* mRNA, YAP1 protein, and target gene expression at 32 hours following sHx, sHx plus *αAhsa1‐siRNA*, and sHx plus *αYap1‐siRNA*. (B) *Yap1* mRNA, YAP1 protein, and target gene expression at 48 hours after sHx and *siRNA* treatment. Note the upregulation for three out of four YAP targets with *αYap1‐siRNA* treatment N = 3/group, *t* test, *P < 0.05. (C) LW/BW and 7‐day survival in *αAhsa1‐siRNA*‐ and *αYap1‐siRNA*‐treated mice after sHx. N = 5/group, *t* test, *P < 0.05. Macroscopic liver appearance at 48 hours is shown to the right

To explore the contribution of YAP1 to the major parenchymal growth phase during regeneration, we performed a knockdown experiment. α*Ahsa1*‐ (hepatic control) and α*Yap1*‐siRNA were intravenously injected 48 hours prior to sHx using a nanoformulation that preferentially targets hepatocytes.^3^, ^15^ Knockdown was effective at 32 hours post‐sHx, as demonstrated through significantly reduced levels of *Yap1* mRNA, YAP1 protein, and of its transcriptional targets *Ctgf*,* Birc5*, and *Myc* (Figure 2A). Importantly, knockdown was limited to hepatocytes, sparing non‐parenchymal liver cells and extrahepatic tissues such as colon (Supplementary Figures 1, 2, 3), confirming the selectivity of the formulation. Similarly, knockdown was effective at 48 hours post‐sHx, with reductions in both *Yap1* and hepatocellular YAP1 akin to 32 hours (Figure 2B, Supplementary Figures 1, 2, 3). Notably, however, the transcriptional targets of YAP1 were upregulated at 48 hours post‐sHx in α*Yap1*‐siRNA‐treated mice relative to α*Ahsa1*‐siRNA‐treated or untreated mice (Figure 2B), suggesting YAP1 is no more the main transcriptional regulator of investigated target genes at this time in the regenerating liver. Of further note, neither histology nor serum markers indicated ponderable liver injury with the knockdown, suggesting no confounding damage is present (Supplementary Figure S4).

**Figure 3:**
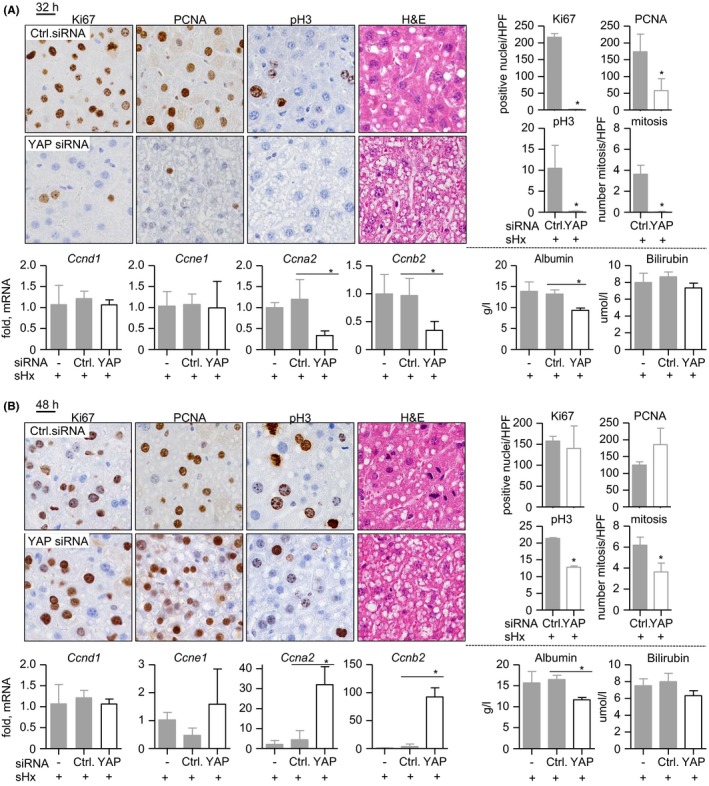
Proliferative and metabolic parameters following knockdown and sHx. (A) Top: immunohistochemistry for Ki67, PCNA, pH3, and histological (H&E) steatosis (Ki67/PCNA/pH3/mitotic counts to the right). Bottom: cyclin gene expression, serum albumin, and serum bilirubin; all at 32 hours following sHx and siRNA treatment. (B) The same parameters as for A at 48 hours following sHx and siRNA treatment. Note the attenuation of effects in the proliferative parameters and the upregulation of *Ccna2*/*b2* in *αYap1‐siRNA*‐treated mice. N = 5/group, *t* test, *P < 0.05

To determine the effects of YAP1 deficiency on the regenerative process, we measured the liver weight gain (LW/BW) after siRNA treatment and resection. Interestingly, LW/BW tended to be reduced (*P* = 0.086) already at hepatectomy in α*Yap1*‐siRNA‐ relative to α*Ahsa1*‐siRNA‐treated mice (Figure 2C), consistent with the role of YAP1 as a physiological regulator of resting liver size.^5^ Remarkably, liver weight gain was completely suppressed up to 32 hours after sHx; at 48 hours, liver weight still was reduced compared to controls, however, the regain was accelerated toward 48 hours in mice with *Yap1* knockdown compared to controls (Figure 2C). Therefore, YAP1 is required for early regeneration, but appears to be dispensable for later liver growth, consistent with the dissociation of nuclear YAP1 from its transcriptional targets at 48 hours post‐sHx (Figures 1C and 2B). Nonetheless, 1‐week survival (ie, the best measure for a functional liver recovery) appeared to be slightly compromised (*P *= 0.09) after *Yap1* knockdown and hepatectomy (Figure 2C), suggesting that the early promotion of liver weight gain (ie, up to 32 hours) through YAP1 is vital to regeneration after tissue loss. Finally, the macroscopic appearance of α*Yap1*‐siRNA‐treated liver was pale at 48 hours after sHx (Figure 2C), pointing to persisting steatosis that typifies liver remnants with a regenerative deficiency.^2^, ^3^, ^16^

### 3.3 YAP1 knockdown affects proliferative markers and cell cycle molecules at 32 hours but not 48 hours after hepatectomy

To further characterize the regenerative deficits following *Yap1* knockdown, we assessed parameters of proliferation and liver function.

Relative to control siRNA, *Yap1* knockdown prior to sHx resulted in a suppression of hepatocytes positive for Ki67 (marking cycling cells) pH3 (G2/M‐phase cells), or for histological mitosis, while PCNA counts (S‐phase cells) were strongly reduced at 32 hours, the usual S‐phase peak after sHx (Figure 3A). Furthermore, histological steatosis was pronounced upon *Yap1* knockdown (Figure 3A, Supplementary Figure S4), consistent with the macroscopic liver appearance (Figure 2C). The expression of *Ccnd1* and *Ccne1* was unaffected; however, both *Ccna2* and *Ccnb2* were markedly downregulated through α*Yap1*‐siRNA (Figure 3A). Serum albumin was reduced, while bilirubin levels remained unchanged following *Yap1* knockdown (Figure 3A), indicating some liver dysfunction with YAP1 deficiency.

At 48 hours (the usual M‐phase peak) after hepatectomy, Ki67 counts were similar and PCNA counts tended (*P* = 0.1) to increase in α*Yap1*‐siRNA‐ vs α*Ahsa1*‐siRNA‐treated mice, while pH3 positivity and mitotic counts were reduced, but no more suppressed as at 32 hours, in *Yap1*‐deficient liver (Figure 3B). These findings suggest that YAP1 deficiency causes an initial proliferative block (eg, evident at 32 hours), which is followed by tardive, compensatory proliferation of hepatocytes independent of YAP1 at 48 hours post‐resection. In agreement, *Ccna2* and *Ccnb2* expression were upregulated (Figure 3B) despite YAP1 knockdown at 48 hours (Figure 2B), while *Ccnd1*/*Ccne1* expression stayed unaffected. Histological steatosis remained more pronounced and albumin reduced in α*Yap1*‐siRNA‐treated animals, reflecting an impaired function—likely because of the still smaller remnant relative to controls at 48 hours (Figure 2C).

Based on the findings shown in Figures 2 and 3, we conclude that YAP1 is essential for hepatocytes to enter the cell cycle and progress through the S‐phase (ie, 32 hours). For further progression (ie, 48 hours), YAP1 is no more needed, either due to compensatory mechanisms elicited through the *Yap1* knockdown, or because its physiological role is limited to the G_0_ > G_1_ > S‐promotion during liver regeneration.

### 3.4 YAP1 is downregulated during earlier, but not later stages of resection‐induced liver failure

To get better insight into the physiological role of YAP1 during liver regeneration, we examined its expression after extended hepatectomy (eHx). Liver failure after eHx develops due to deficient progression through the hepatocellular S‐ and M‐phases associated with a shutdown of pro‐proliferative pathways.^2^, ^3^, ^16^ If YAP1 indeed functions in the early cell cycle (including the S‐phase) but is dispensable for the M‐phase, its activity should be impaired at 32 hours, but not 48 hours, after eHx.

**Figure 4:**
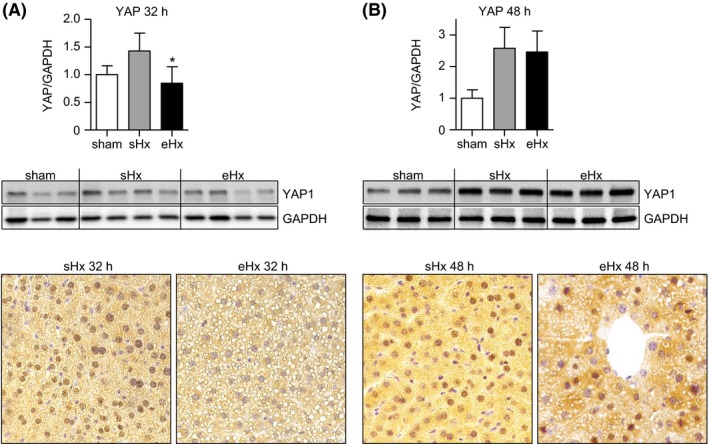
YAP1 protein expression during resection‐induced liver failure. Quantified YAP1 expression, corresponding immunoblots, and YAP1 immunohistochemistry at 32 hours (A) and 48 hours (B) after sHx and eHx. eHx was compared to sHx, revealing a significant downregulation of YAP1 at 32 hours, but not at 48 after eHx. Note the low nuclear expression of YAP1 at 32 hours after eHx. The differences between sham and sHx at 32 hours and sHx/eHx at 48 hours were significant as well (see Figure 1B). N = 5/group, *t* test, *P < 0.05

When comparing sHx vs eHx relative to sham surgery, YAP1 protein expression was elevated at 32 hours after sHx, but remained at sham levels after eHx (Figure 4A). Likewise, YAP1 nuclear accumulation was reduced at 32 hours after eHx relative to sHx (Figure 4A). YAP1 thus is not upregulated when S‐phase progression is defective, concordant with a role in promoting the earlier cell cycle stages (Figures 2C and 3A). At 48 hours, however, YAP1 protein was similarly elevated and displayed comparable nuclear accumulation after both sHx and eHx (Figure 4B). Given that transient arrest at the M‐phase is the most defining cell cycle defect in resection‐induced liver failure,^2^, ^3^, ^16^ nuclear YAP1 cannot be associated with the regulation of the M‐phase in these settings. This finding is consistent with the reprise of liver weight gain (Figure 2C) and proliferative activity (Figure 3B) seen at 48 hours post‐sHx despite YAP1 knockdown (Figure 2B). Therefore, compensatory mechanisms triggered through the acute YAP1 deficiency are unlikely causal in dissociating YAP1 activity from regeneration at 48 hours post‐sHx. Rather, YAP1's key physiological role in liver regeneration is the initial promotion of the hepatocyte cycle to and through the S‐phase, while the M‐phase may be governed through other pathways with redundant downstream targets.

### 3.5 YAP1 is induced in human liver regeneration

**Figure 5:**
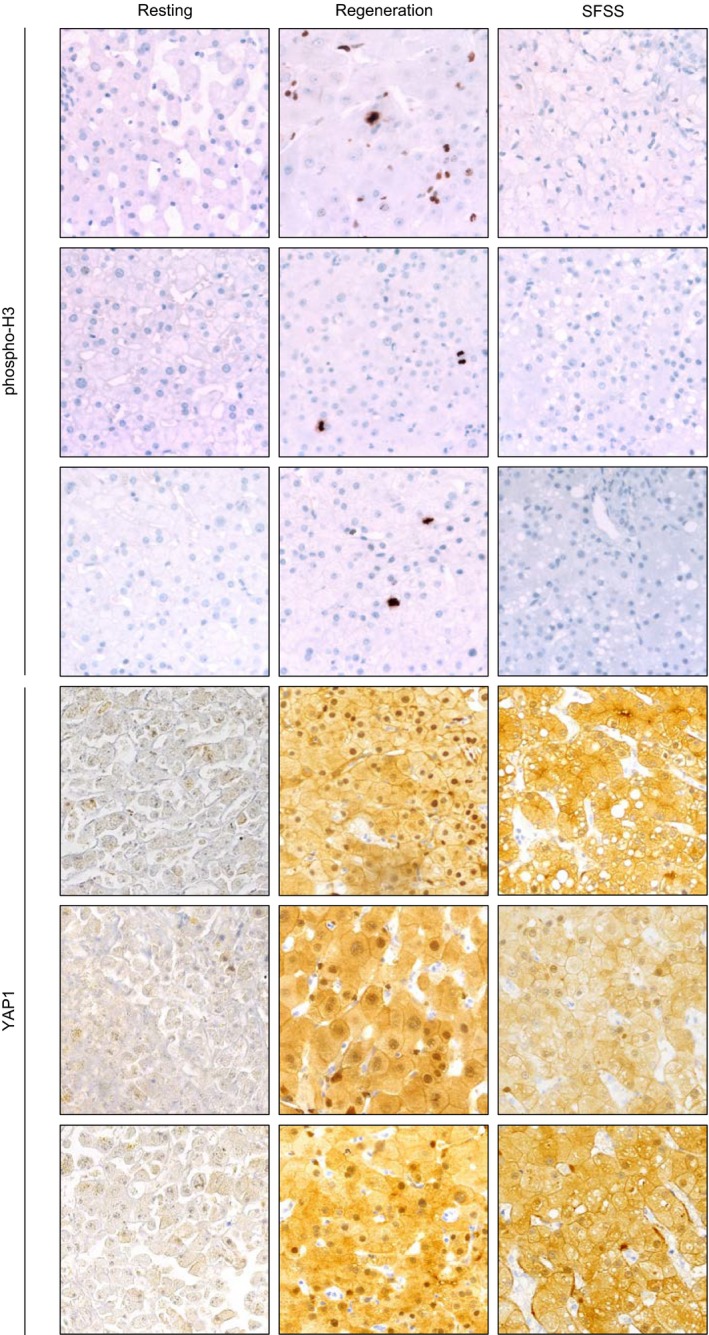
YAP1 immunohistochemistry on human liver tissue. Representative examples of resting, regenerating, and SFSS liver each of three different patients (A, B, and C) are shown. Immunochemistry for pH3 was included to illustrate the regenerative state. See Methods for patient characteristics. Although data on the time of biopsy retrieval were available, no associations with specific cell cycle phases were possible, as cell cycle progression is heterogeneous and strongly dependent on the disease background and the state of patients' livers

To validate a physiological role of YAP1 in liver regeneration, we examined biopsy material retrieved from human liver before and after hepatectomy. Biopsies were available from patients (n = 7, 7‐11 days postsurgery) that recovered from hepatectomy without complications (ie, successful regeneration) and from patients (n = 7, 5‐14 days postsurgery) that developed the SFSS (ie, resection‐induced liver failure). We previously have shown that human SFSS features similar characteristics as its mouse counterpart, namely deficient cell cycle progression, downregulation of the cell cycle promoter FOXM1, upregulation of P21, metabolic insufficiency (persisting steatosis, hypoalbuminemia, hyperbilirubinemia), and an elevated mortality.^2^, ^3^ When examining YAP1 by immunohistochemistry, the protein displayed little expression in resting liver before hepatectomy, but was markedly upregulated (including nuclear expression) in regenerating human liver (Figure 5). In contrast, YAP1 nuclear expression was reduced in human SFSS liver relative to regenerating liver (Figure 5). No information, however, was available on the particular cell cycle phases of investigated human livers. Nonetheless, these findings strongly suggest that YAP1 contributes to the successful regeneration of human liver after hepatectomy.

## 4 DISCUSSION

Given the role of YAP1 in organ size control, a function for the transcriptional coactivator in liver regeneration has been anticipated. Available data support this view,^7^, ^8^, ^9^, ^10^, ^11^, ^12^ however, the need of YAP1 for the liver to regenerate has remained ill‐proven. Here, we show that liver regeneration in mice is stalled for the first 32 hours after tissue loss when YAP1 is inhibited; however, liver weight regain is resumed thereafter. The pro‐regenerative role of YAP1 is thus confined to the progression of hepatocytes into cycle and through the S‐phase, but is expendable for a further progression through the M‐phase. Such a role is reflected in resection‐induced liver failure with deficient S‐ and M‐phase progression, where YAP1 fails to be induced during the S‐phase peak, but is upregulated during the M‐phase. Finally, YAP1 may have a similar function in human liver regeneration, as evinced through the examination of clinical samples.

Confirming its peak expression at 32‐48 hours after hepatectomy,^8^, ^10^ we chose a knockdown approach to probe YAP1's function. Acute deficiency may avoid compensation through other proteins such as YAP1's partner TAZ, which is upregulated in YAP1 knockout liver.^13^ Knockdown was effective during the expression peak at both the mRNA and protein level. However, as a limitation, we cannot exclude off‐target effects even though the siRNA used was selected among 12 sequences designed to have no homology to other genomic sequences (Axolabs Gmbh). On the other hand, no systemic or cell‐unspecific effects are expected, as siRNA was delivered through a nanoformulation that we confirmed to specifically target hepatocytes (Figures S1‐S3).^3^, ^15^

Our findings of YAP1 as a promoter of the early hepatocyte cell cycle are consistent with its generic function in proliferation. Following mechanical tension or the loss of cell‐cell contacts, YAP1 fosters cell cycle entry and progression through the S‐phase.^17^, ^18^ Although YAP1's cell cycle functions may be divergent,^19^ the regulation up to the S‐phase is a recurring feature.^20^ The defects in cell cycle entry (Ki67) and the S‐phase (*Ccna2*, PCNA) upon knockdown conceivably explain the lack of liver weight regain up to 32 hours after sHx. However, *Ccnd1/e1* expression was unaffected, perhaps implying YAP1 regulates cell cycle entry in regenerating liver via other molecules such as the E2F‐dependent transcriptional network.^21^

Contrary to our findings, Lu et  al^12^ have observed only slight impairment of early regeneration in mouse liver with constitutive YAP1/TAZ deletion, while major defects were noted between 7 and 14 days after sHx. These double‐knockout‐livers, however, displayed several chronic pathologies, including ill‐developed bile ducts, inflammation, parenchymal injury, hepatomegaly, and neoplastic changes. Therefore, the elevated basal proliferation seen in YAP1/TAZ^−/−^ livers may have masked early regenerative defects, while the pre‐existing parenchymal injury may have exaggerated regenerative deficiencies over time. An alternative explanation for the divergent outcomes may be the concomitant loss of TAZ in their model.^12^ In another recent report, liver‐specific *Yap1* knockout (induced by adenoviral Cre) has resulted in reduced Ki67 counts (by ~50%) at 48 hours post‐hepatectomy.^11^ We could not confirm this finding, possibly because the knockout strategy was more efficient than our knockdown in reducing YAP1 levels, leading to a more severe initial delay that left more traces at 48 hours post‐sHx. Comparisons with the *Yap1* knockout model however remain difficult, because only one marker at one time point has been assessed in the latter.^11^ Anyhow, our approach of acute deficiency induced prior to hepatectomy demonstrates that YAP1’s specific contribution is vital for early but dispensable for later stages of liver regeneration, a finding corroborated with our liver failure model.

Intriguingly, Ki67, PCNA, cyclins and YAP targets were re‐elevated at the mitotic peak (48 hours) after sHx despite effective knockdown, suggesting a contribution of other pro‐regenerative transcriptional activators. β‐catenin, for example, is thought to be inhibited by YAP1 in the liver,^5^ promotes regeneration, and can likewise activate the YAP1 targets *Birc5*,* Ctgf, Cyr61*, and *Myc* in hepatic cells.^22^, ^23^, ^24^, ^25^ Indeed, we observed increased nuclear β‐catenin expression upon YAP1 knockdown at 48 hours after sHx (Supplementary Figure S5), consistent with a reduced inhibition of β‐catenin^5^ leading to increased promotion of the transcriptional targets shared with YAP1. The upregulation of β‐catenin in response to YAP1 deficiency perhaps reflects the redundant mitogenic pathways that ensure recovery also under suboptimal conditions—a key principle behind liver regeneration.^26^

In keeping with the above, our model of resection‐induced liver failure was associated with a re‐elevation of YAP1 at 48 hours despite its defective induction at 32 hours. Liver fails after eHx particularly because of a p21‐dependent arrest before mitosis, with compensatory regeneration starting not before 24 hours later.^2^ Therefore, the upregulation at 48 hours after eHx confirms the dissociation of YAP1 from mitotic progression during liver regeneration. Notably, the inhibition of both S‐phase and M‐phase progression prior to sHx induces full SFSS symptoms (steatosis, hypoalbuminemia, hyperbilirubinemia, increased mortality) akin to eHx.^3^ In contrast, sHx in YAP1‐deficient mice caused only mild SFSS (normal bilirubin, mild steatosis, slightly reduced mortality), again entirely consistent with a defect limited to the S‐phase. If not promoting tissue growth at 48 hours post‐sHx, why is YAP1 upregulated then? Metabolic functions of YAP1^5^ perhaps might provide a conjectural explanation: recently, YAP1 has been reported to promote glycolysis,^27^, ^28^ a function that may contribute to the switch from lipids to glucose as a preferential energy source observed after 48 hours in the regenerating liver.^16^ Alternatively, YAP1 may contribute to the remodeling of the extracellular matrix, an obligate event in regenerating liver.^26^ Recent data indicate YAP1 promotes the acquisition of mesenchymal traits, enabling regenerating hepatocytes to produce fibrous matrix particularly at later times after hepatectomy^11^—a potential contribution to the reconstitution of the extracellular liver environment.

In summary, we report an essential role for YAP1 in promoting hepatocyte cell cycle progression to and through the S‐phase in regenerating liver after tissue loss. Our findings further suggest additional, however, non‐proliferative, functions of YAP1 during later regenerative phases when hepatocyte mitosis peaks. These observations were corroborated in regenerative failure typified through deficient S‐ and M‐phase progression, where YAP1 is downregulated only at the S‐ but not the M‐phase peak. Akin to the mouse liver, YAP1 was induced in human livers with successful regeneration, but not in those with failed regeneration, suggesting YAP1's role in liver regeneration is conserved in mammals. Future research should address whether pharmacological YAP1 activators currently in development^29^ may bear potential to aid liver regeneration in the clinic.

## CONFLICT OF INTEREST

None of the authors has any conflict of interest to report.

## AUTHOR CONTRIBUTION

C. Tschuor, E. Kachaylo, U. Ungethüm, Z. Song, B. Humar, P. Sánchez‐Velázquez, M. Linecker, P. Kambakamba, D.A. Raptis, P. Limani, and D. Eshmuminov performed the experiments and analyzed the data; B. Humar, E. Kachaylo, and C. Tschuor interpreted the data; C. Tschuor, E. Kachaylo, B. Humar, K. Lehmann, and R. Graf designed the experiments; B. Humar conceived and designed the study; B. Humar and C. Tschuor drafted the manuscript; B. Humar, E. Kachaylo, A. Colombano, and P.A. Clavien critically revised the manuscript; B. Humar wrote the final version; P.A. Clavien, B. Humar, R. Graf, and C. Tschuor organized the funding; all authors approved the final version.
