# Supplementary material for: Yes‐associated protein promotes early hepatocyte cell cycle progression in regenerating liver after tissue loss
Source: FASEB Bioadv. 2018 Nov 27;1(1):51–61. doi: 10.1096/fba.1023 (PMC6351850; doi:10.1096/fba.1023)

Supplementary Material

Yes-associated protein promotes early hepatocellular cell cycle progression in regenerating liver after tissue loss

Christoph Tschuor*, Ekaterina Kachaylo*, Udo Ungethüm, Zhoulun Song, Kuno Lehmann, Patricia Sánchez-Velázquez, Michael Linecker, Patryk Kambakamba, Dimitri A. Raptis, Perparim Limani, Dilmurodjon Eshmuminov, Rolf Graf, Amedeo Columbano, Bostjan Humar#, Pierre-Alan Clavien#

*shared first authorship

#shared senior authorship

- Supplementary Figures

Supplementary Figures

Supplementary Figure 1

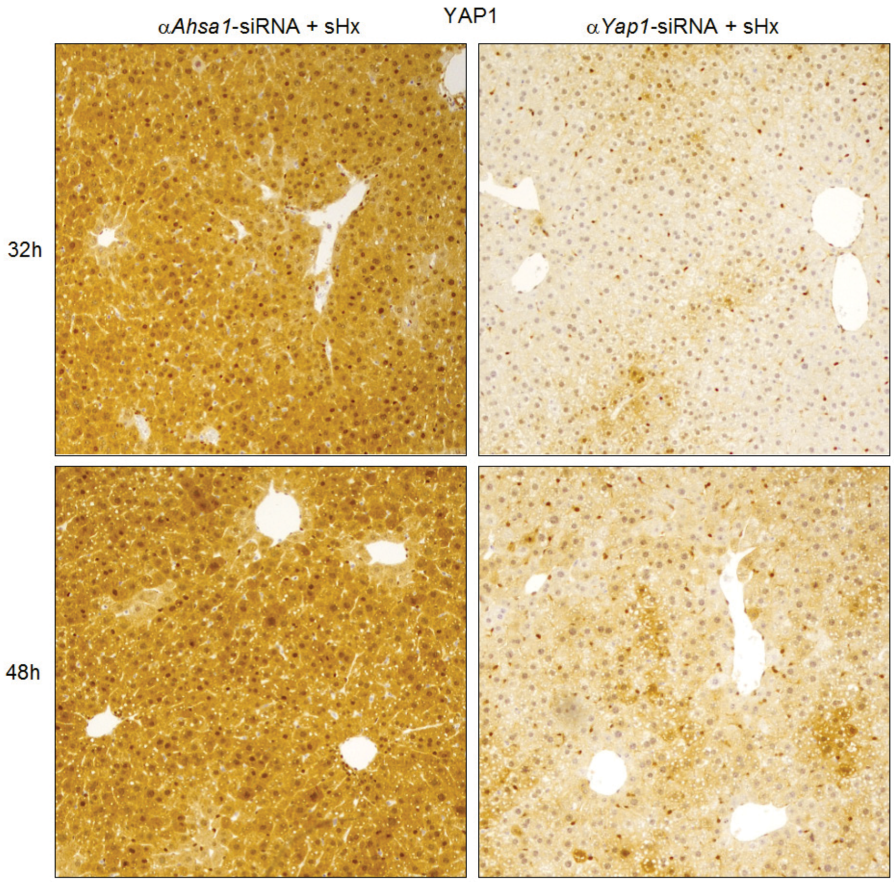

Figure S1. YAP1 immunohistochemistry confirms YAP1 protein knockdown after siRNA treatment. Control (α*Ahsa1*-siRNA) and α*Yap1*-siRNA-treated livers are shown at 32h and 48h post sHx. Note the global reductions in both cytoplasmic and nuclear YAP1 with the knockdown. Magnification 10x.

Supplementary Figure 2

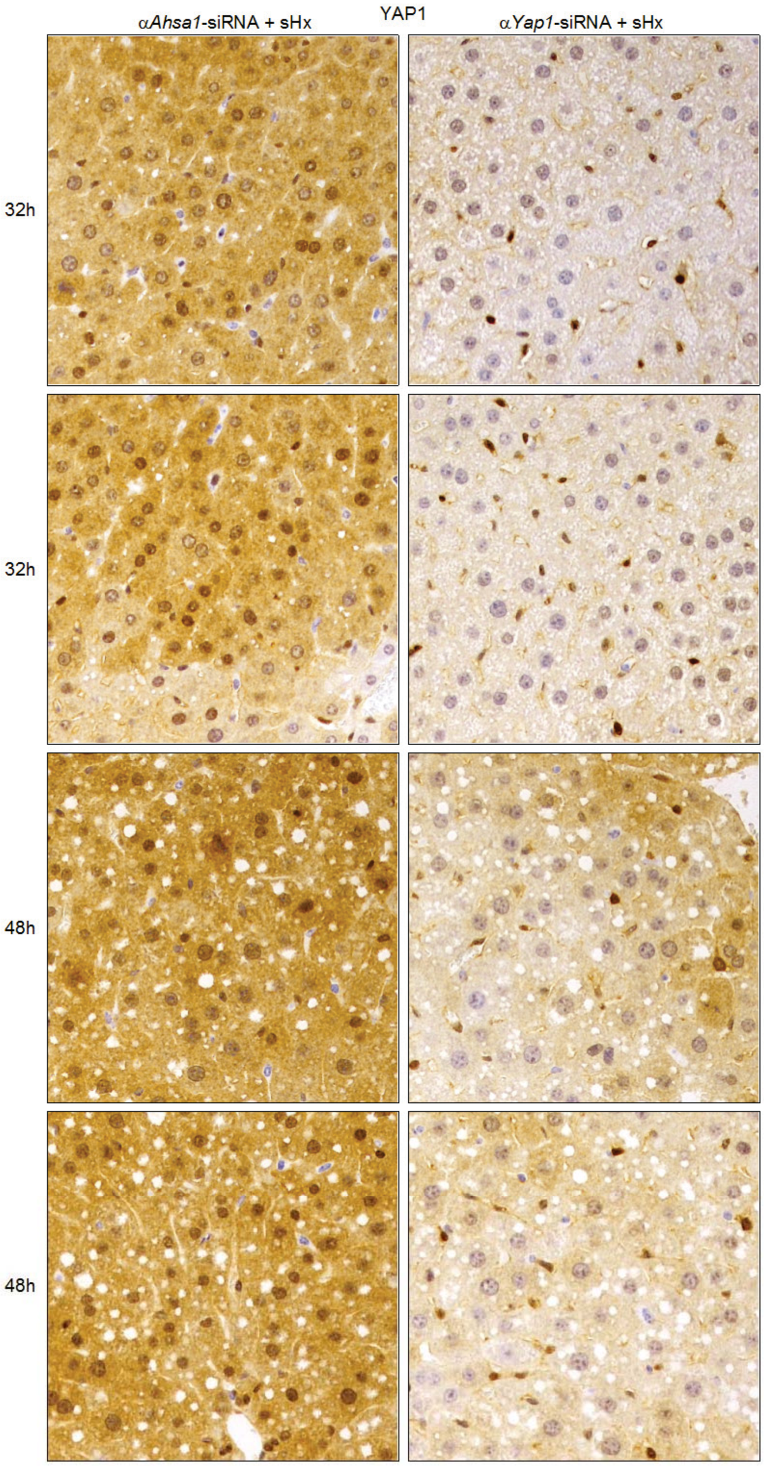

Figure S2. YAP1 knockdown affects hepatocytes, but spares non-parenchymal liver cells. Two images are shown for each treatment (α*Ahsa1*-siRNA *vs*. α*Yap1*-siRNA) and time point post sHx (32h, 48h). Note that the strong nuclear signal in non-parenchymal cells is not affected by the knockdown, unlike for hepatocytes. Magnification 40x.

Supplementary Figure 3

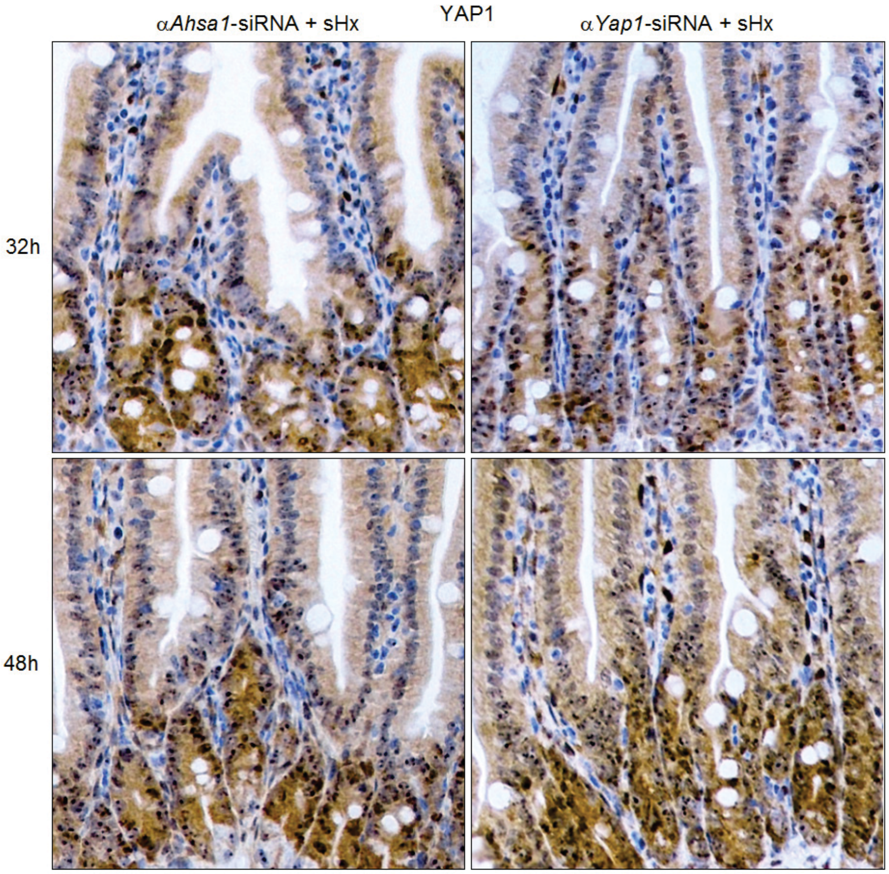

Figure S3. YAP1 knockdown does not affect extrahepatic tissues. The proliferating region of intestinal crypts displays nuclear expression of YAP1, which remains unaltered following sHx and pre-treatment with either α*Ahsa1*-siRNA or α*Yap1*-siRNA. siRNA was delivered by a nanoformulation from Axolabs Gmbh specifically designed to target hepatocytes. Magnification 20x.

Supplementary Figure 4

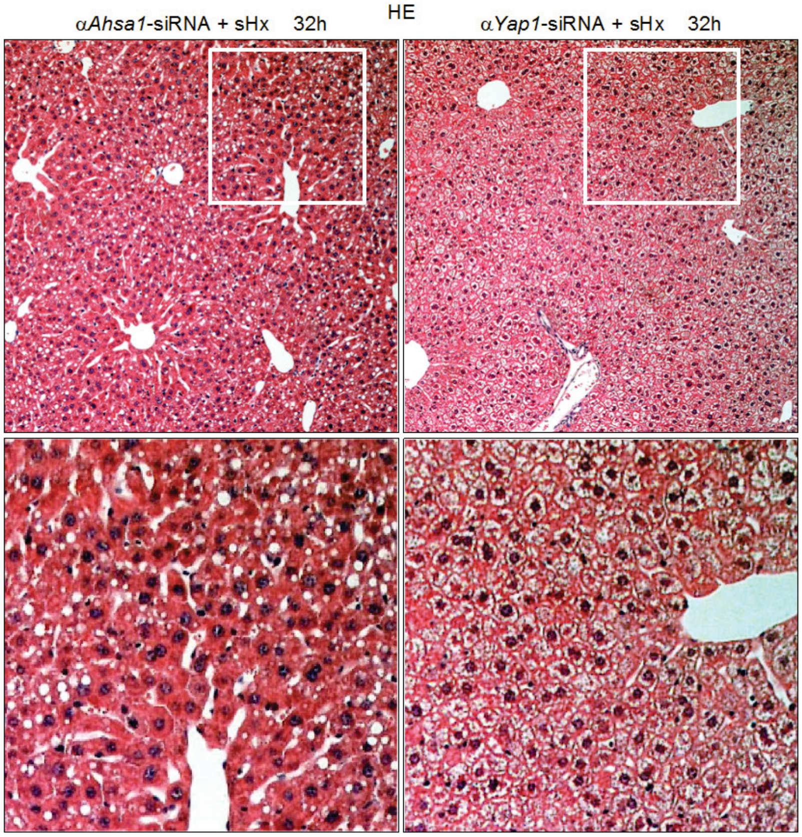

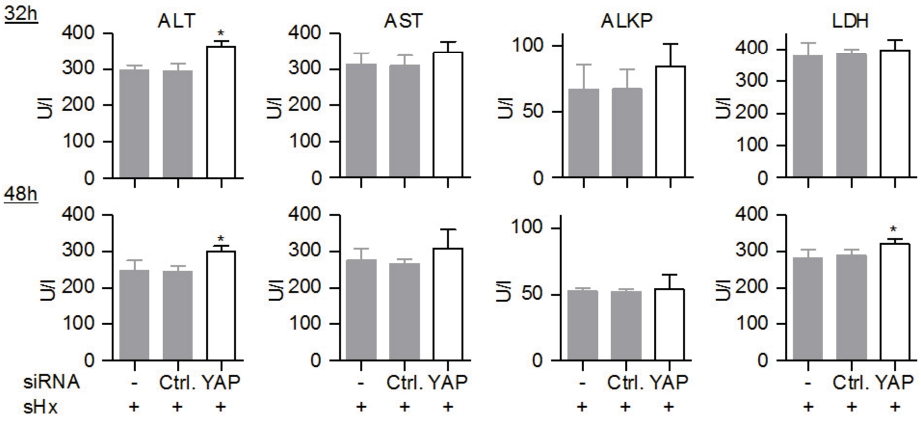

Figure S4. Liver histology and serum liver markers after sHx and siRNA-pre-treatment. Apart from pronounced steatosis, liver histology appears normal in α*Yap1*-siRNA-treated relative to α*Ahsa1*-siRNA-treated mice at 32h after sHx. Magnified squares of upper images (10x magnification) are shown below. Bottom: serum levels of injury markers AST, ALT, ALKP and LDH and ALKP at 32h and 48h after sHx, α*Ahsa1*-siRNA plus sHx, and α*Yap1*-siRNA plus sHx. *Yap1* knockdown is associated with minimal elevations in some of the injury markers, however at very low absolute levels, indicating the absence of substantial liver injury. N=5/group, t-test, *P<0.05.

Supplementary Figure 5

Figure S5. Immunofluorescence for β-catenin on liver after siRNA treatment and standard hepatectomy. Immunofluorescence reveals mebraneous and nuclear expression of β-catenin in the regenerating liver. Although the difference in nuclear positivity are not obvious at a first glance, counting of hepatocytes with nuclear β-catenin expression (numbers ±SD in upper left corner) suggests an increase in β-catenin activity at 48h after sHx and pretreatment with α*Yap1*-siRNA relative to control siRNA. YAP1 is known to oppose β-catenin activities in the liver. Its knockdown may hence enhance nuclear expression of β-catenin expression, which can promote the expression of target genes shared with YAP1 (i.e. *Ctgf*, *Birc5*, *Cyr61*, *Myc*). β-catenin antibody (Cell Signaling, Beverly, MA, USA; 9582) was visualized with an AlexaFluor488 secondary antibody (Fisher Scientific, Illkirch, France, A-11008). DAPI was used for nuclear staining.

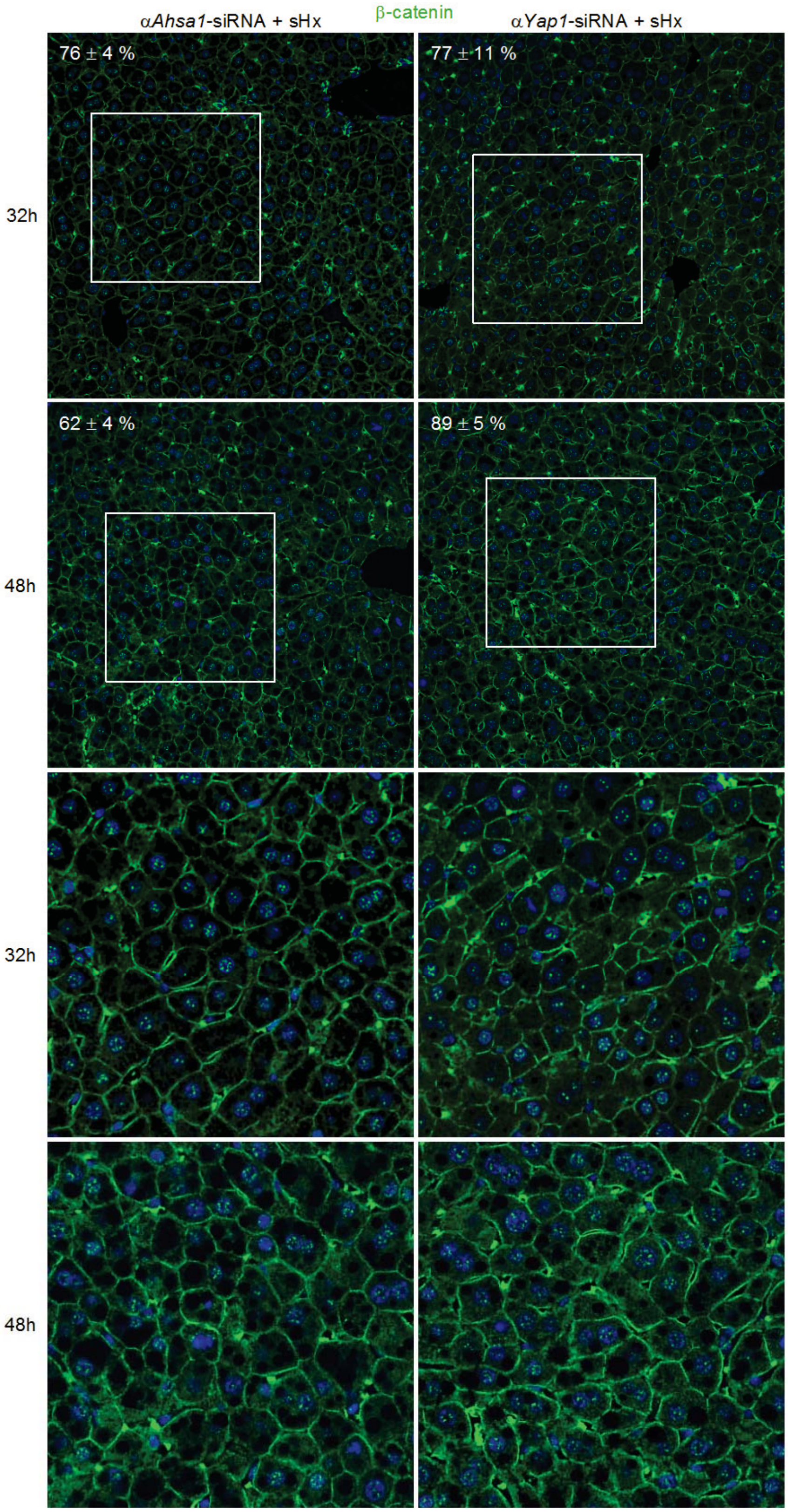

Supplement: Supplementary file 1 [file FBA2-1-51-s001.pdf]
